# Clinical characteristics and risk factors of fatal patients with COVID-19: a retrospective cohort study in Wuhan, China

**DOI:** 10.1186/s12879-021-06585-8

**Published:** 2021-09-14

**Authors:** Meng Jin, Zequn Lu, Xu Zhang, Yanan Wang, Jing Wang, Yimin Cai, Kunming Tian, Zezhong Xiong, Qiang Zhong, Xiao Ran, Chunguang Yang, Xing Zeng, Lu Wang, Yao Li, Shanshan Zhang, Tianyi Dong, Xinying Yue, Heng Li, Bo Liu, Xin Chen, Hongyuan Cui, Jirong Qi, Haining Fan, Haixia Li, Xiang-Ping Yang, Zhiquan Hu, Shaogang Wang, Jun Xiao, Ying Wang, Jianbo Tian, Zhihua Wang

**Affiliations:** 1 Department of Respiratory and Critical Care Medicine, Renmin Hospital of Wuhan University, Wuhan, Hubei China; 2 Department of Epidemiology and Biostatistics, Key Laboratory for Environment and Health, School of Public Health, Tongji Medical College, Huazhong University of Sciences and Technology, No. 13, Hangkong Rd, Wuhan, 430030 Hubei China; 3 Key Laboratory of Environment and Health, Ministry of Education & Ministry of Environmental Protection, and State Key Laboratory of Environmental Health (Incubating), School of Public Health, Tongji Medical College, Huazhong University of Science and Technology, Wuhan, Hubei China; 4 Department of Urology, Tongji Hospital, Tongji Medical College, Huazhong University of Science and Technology, No. 1095, Jiefang Ave, Wuhan, 430030 Hubei China; 5 Institute of Reproductive Health, Tongji Medical College, Huazhong University of Science and Technology, Wuhan, Hubei China; 6 Department of Critical Care Medicine, Tongji Hospital, Tongji Medical College, Huazhong University of Science and Technology, Wuhan, Hubei China; 7 Department of Oncology, Tongji Hospital, Tongji Medical College, Huazhong University of Science and Technology, Wuhan, Hubei China; 8 Department of General Surgery, Beijing Hospital, National Center of Gerontology, Institute of Geriatric Medicine, Chinese Academy of Medical Sciences, Beijing, China; 9 Department of Cardiothoracic Surgery, Nanjing Children’s Hospital Affiliated to Nanjing Medical University, Nanjing, Jiangsu China; 10 Department of General Surgery, Qinghai University Affiliated Hospital, Xining, Qinghai China; 11 Computer Center, Tongji Hospital, Tongji Medical College, Huazhong University of Science and Technology, Wuhan, Hubei China; 12 Department of Immunology, Tongji Medical College, Huazhong University of Science and Technology, Wuhan, Hubei China; 13 Department of Virology, Wuhan Centers for Disease Prevention and Control, Wuhan, Hubei China; 14 School of Health Sciences, Wuhan University, Wuhan, 430071 China

**Keywords:** COVID-19, Death risk, Risk prediction models, Immune cells subsets

## Abstract

**Background:**

The coronavirus disease 2019 (COVID-19) has caused a global pandemic, resulting in considerable mortality. The risk factors, clinical treatments, especially comprehensive risk models for COVID-19 death are urgently warranted.

**Methods:**

In this retrospective study, 281 non-survivors and 712 survivors with propensity score matching by age, sex, and comorbidities were enrolled from January 13, 2020 to March 31, 2020.

**Results:**

Higher SOFA, qSOFA, APACHE II and SIRS scores, hypoxia, elevated inflammatory cytokines, multi-organ dysfunction, decreased immune cell subsets, and complications were significantly associated with the higher COVID-19 death risk. In addition to traditional predictors for death risk, including APACHE II (AUC = 0.83), SIRS (AUC = 0.75), SOFA (AUC = 0.70) and qSOFA scores (AUC = 0.61), another four prediction models that included immune cells subsets (AUC = 0.90), multiple organ damage biomarkers (AUC = 0.89), complications (AUC = 0.88) and inflammatory-related indexes (AUC = 0.75) were established. Additionally, the predictive accuracy of combining these risk factors (AUC = 0.950) was also significantly higher than that of each risk group alone, which was significant for early clinical management for COVID-19.

**Conclusions:**

The potential risk factors could help to predict the clinical prognosis of COVID-19 patients at an early stage. The combined model might be more suitable for the death risk evaluation of COVID-19.

**Supplementary Information:**

The online version contains supplementary material available at 10.1186/s12879-021-06585-8.

## Background

The global spread of coronavirus disease 2019 (COVID-19) has become a major global public health issue. The COVID-19 pandemic was sweeping across borders, sickening and killing people in nearly every country. Until January 5, 2021, a total of 84,233,579 cases were confirmed and the death numbers of COVID-19 attained 1,843,293 [1] currently. Considering the adverse effects of COVID-19 and its high mortality rate, it is urgent for us to understand the risk factors that influence the incidence of death and develop a comprehensive risk model evaluating the death risk of COVID-19.

Recent evidence has shown that the infection caused by severe acute respiratory syndrome coronavirus 2 (SARS-CoV-2) induces clusters of severe and even fatal pneumonia which had high mortality [2]. Several scores have been considered as good evaluation indexes for sepsis, septic shock, multi-organ dysfunction, and even death in patients suffering from bacterial or viral pathogen infection, such as the sequential organ failure assessment (SOFA), quick SOFA (qSOFA), acute physiology, chronic health evaluation II (APACHE II) and systemic inflammatory response syndrome (SIRS) scores [3–8]. Besides, it has been reported that the pro-inflammatory cytokine IL-6 and organ damage biomarkers, such as D-dimer and NT-proBNP were risk factors for death of adults with COVID-19 [6, 9, 10]. Some case series have reported the clinical characteristics or potential risk factors for fatal patients with COVID-19 [6, 9, 11]. However, the sample sizes in most of these reports were limited and detailed information about biochemical biomarkers dynamics, immune cell subsets or pertained treatments of fatal COVID-19 patients have not yet been well described. Most importantly, no researches have been done to develop a comprehensive prediction model integrating these scores in the current, inflammatory indices, immune cells subsets and organ damage risk factors for death risk of COVID-19, which is of great value for better risk stratification, death-related biomarkers identification and clinical treatments strategies-making.

Here we performed a retrospective, observational study in fatal COVID-19 patients and presented the details of patients with definite clinical outcome (death or discharge) from Tongji Hospital, Huazhong University of Science and Technology (HUST), which is the biggest designated hospital for treating severely or critically ill COVID-19 patients in Wuhan, an epicenter of COVID-19 outbreak. The objective of this study was to explore potential risk factors of death for COVID-19 and establish a comprehensive risk model evaluating the death risk of COVID-19, which is of utmost importance for early clinical management and successful establishment of standardized treatment protocols, ultimately curbing the rising fatality rate of COVID-19.

## Methods

### Study design and participants

This retrospective cohort study was performed in patients with confirmed COVID-19 from Tongji Hospital, Tongji Medical College, HUST, a designated hospital for severely or critically ill COVID-19 patients. All fatal patients with COVID-19 (*N* = 281) were enrolled from January 13 to March 31, 2020. Subsequently, 712 survivors were statistically matched by propensity score matching [12] at an approximate ratio of 3:1 based on age, sex and comorbidities. This study was approved by the Ethics Committee of Tongji Hospital, Tongji Medical College, HUST and granted a waiver of informed consent from study participants.

### Data collection

Epidemiological, clinical, radiological, laboratory, clinical treatments, and clinical outcomes data of all patients with laboratory-confirmed SARS-CoV-2 were obtained with data collection forms from electronic medical records of Tongji Hospital. The researches on other topics [9, 13] may obtain some information of non-survivors in Tongji hospital, however, our study collected more information about cases and detailed data. The admission and in-hospital data of these patients were collected, reviewed and verified by a trained team of physicians. Any missing or uncertain records were collected and clarified through communication with involved health-care providers and their families. The detailed and standardized information of demographic data, underlying comorbidities, initial symptoms, vital signs, and chest computed tomographic (CT) were recorded or diagnosed at hospital admission. The complications, treatments, clinical outcomes (survivors and non-survivors) and hospital length of stay were monitored to March 31, 2020, the final data of follow-up. Laboratory examinations including blood routine, immune cells subsets, inflammatory cytokines and biomarkers, blood gas assay, cardiac function test, renal function test, liver function test, pancreatic function test, coagulation test, and metabolism test were detected on the first diagnosed date. The time of follow-up was defined as the duration from admission to outcomes (survivor/non-survivor) of patients. There were no cases lost to follow-up in this study attributed to standardized government managements and close tracking for COVID-19 pandemic.

### Definitions

The detailed definitions of complications including acute respiratory distress syndrome (ARDS), acute pancreatic injury, acute liver damage, acute cardiac injury, disseminated intravascular coagulation (DIC), SOFA, qSOFA, APACHE II and SIRS scores were listed in the Additional file 1: Methods.

### Statistical analysis

Continuous variables were presented as median and interquartile range (IQR) or mean and standard deviation (SD). Categorical variables were expressed as number (%). For continuous variables, Student’s *t*-test was used for normal distributed data, and Mann–Whitney U non-parameter test was used for non-normal distributed data. The Pearson’s χ^2^ test or Fisher’s exact test were applied for categorical variables. Time to events (recovered or death) was defined as the time from hospital admission to events. The univariate Cox regression models were used to determine hazard ratios (HRs) and 95% confidence intervals (CIs) for the association between individual factors and death risk of COVID-19. The multivariate Cox proportional hazards model analysis was used to establish different comprehensive death risk score models including four score systems, biochemical biomarkers, complications, and a combined system integrating all scores. The significant variables from the univariable analysis of the death risk were considered as the candidates. The stepwise regression was used for the selection of the prediction for the model. Each comprehensive risk score model of candidate variables was established according to the following formula: Risk score (RS) = ∑β variables × Expvariables. The β variables represented the estimated contribution coefficient of independent candidate variables in the multivariate Cox regression analysis and Expvariables denoted the level of independent candidate variables. According to this formula, the risk scores of each patient of different death risk score prediction models were computed. The combined system was performed based on the integrating risk scores of different comprehensive models. Then we calculated sensitivity, the specificity of different death risk score prediction models with survival, timeROC packages for all analyses in R version 3.50. Time-dependent receiver operating characteristic (ROC) curves and areas under the curves (AUCs) were used to assess the prognostic accuracy of each model [14]. A two-sided *P* value < 0.05 was considered statistically significant.

## Results

### Clinical characteristics and laboratory findings of non-survivors and survivors with COVID-19

281 (28.30%) non-survivors and 712 (71.70%) survivors with COVID-19 were enrolled in this study. Considering that the age, sex, and comorbidities had been reported to be the common death risk factors of COVID-19, enrolled survivors and non-survivors were statistically matched based on these risk factors. As summarized in Table 1 and Additional file 1: Table S1, the most common symptoms of all patients were fever and cough, non-survivors were more likely to have dyspnea (50.39% vs 42.23%, *P* = 0.026) than survivors. Besides, non-survivors tended to have more abnormal vital signs on admission, such as higher temperature, heart rate and respiratory rate.

**Table 1:** Demographic, clinical, radiographic, laboratory findings of survivors and non-survivors with COVID-19

Indicators	Total	Survivors		Non-survivors		*P* value
	*N* = 993	*N* = 712		*N* = 281		
Characteristics						
Age, years	68(63–74)	*N* = 712	68(63–73)	*N* = 281	69(62–77)	0.100
Sex		*N* = 712		*N* = 281		
Male	655(65.96%)		464(65.17%)		191(67.97%)	0.401
Female	338(34.04%)		248(34.83%)		90(32.03%)	
Comorbidities						
Hypertension	401(40.38%)	*N* = 712	292(41.01%)	*N* = 281	109(38.79%)	0.521
Diabetes	174(17.52%)	*N* = 712	134(18.82%)	*N* = 281	40(14.23%)	0.087
Coronary heart disease	104(10.47%)	*N* = 712	72(10.11%)	*N* = 281	32(11.39%)	0.554
Cerebrovascular disease	39(3.93%)	*N* = 712	27(3.79%)	*N* = 281	12(4.27%)	0.727
Pulmonary tuberculosis	22(2.22%)	*N* = 712	13(1.83%)	*N* = 281	9(3.20%)	0.184
Hepatitis	17(1.71%)	*N* = 712	10(1.40%)	*N* = 281	7(2.49%)	0.234
Chronic bronchitis	19(1.91%)	*N* = 712	11(1.54%)	*N* = 281	8(2.85%)	0.177
Chronic obstructive pulmonary disease	9(0.91%)	*N* = 712	5(0.70%)	*N* = 281	4(1.42%)	0.280
Initial symptoms	*N* = 919		*N* = 663		*N* = 256	
Fever	718(78.13%)		523(78.88%)		195(76.17%)	0.373
Cough	632(68.77%)		471(71.04%)		161(62.89%)	0.017*
Dyspnea	409(44.50%)		280(42.23%)		129(50.39%)	0.026*
Expectoration	399(43.42%)		293(44.19%)		106(41.41%)	0.445
Diarrhoea	199(21.65%)		140(21.12%)		59(23.05%)	0.524
Fatigue	170(18.50%)		119(17.95%)		51(19.92%)	0.490
Chest tightness	147(16.00%)		106(15.99%)		41(16.02%)	0.992
Chills	97(10.55%)		72(10.86%)		25(9.77%)	0.628
Myalgia	82(8.92%)		67(10.11%)		15(5.86%)	0.043*
Anorexia	71(7.73%)		50(7.54%)		21(8.20%)	0.736
Headache	56(6.09%)		43(6.49%)		13(5.08%)	0.424
Vertigo	40(4.35%)		21(3.17%)		19(7.42%)	0.005*
Others^η^	124(13.49%)		104(15.69%)		20(7.81%)	0.002*
Vital signs						
Temperature, ℃	36.6(36.3–37.1)	*N* = 712	36.5(36.2–36.9)	*N* = 281	36.8(36.4–37.6)	< 0.0001*
Heart rate, bpm	92(80–105)	*N* = 712	88(78–99)	*N* = 281	102(90–115)	< 0.0001*
Respiratory rate, bpm	20(20–23)	*N* = 712	20(20–21)	*N* = 281	26(21–33)	< 0.0001*
Mean arterial pressure, mmHg	95(86.67–104.33)	*N* = 711	96.67(89.33–105.67)	*N* = 281	88.67(79–99)	< 0.0001*
CT findings	*N* = 757		*N* = 699		*N* = 58	
Patchy shadows	586(77.41%)		544(77.83%)		42(72.41%)	0.344
Ground-glass opacity	338(44.65%)		307(43.92%)		31(53.45%)	0.161
Fibrous stripes	262(34.61%)		253(36.19%)		9(15.52%)	0.001*
Pleural thickening	212(28.01%)		188(26.90%)		24(41.38%)	0.018*
Nodules	60(7.93%)		55(7.87%)		5(8.62%)	0.800 ^a^
Lymphadenia	214(28.27%)		192(27.47%)		22(37.93%)	0.089
Bilateral pulmonary	735(97.09%)		677(96.85%)		58(100.00%)	0.413 ^a^
Right lung	12(1.59%)		12(1.72%)		0(0.00%)	0.614 ^a^
Left lung	10(1.32%)		10(1.43%)		0(0.00%)	1.000 ^a^
Laboratory examinations						
Blood routine						
Leukocytes, # (*N* = 993), × 10^9^/L	6.12(4.66–8.28)	*N* = 712	5.63(4.43–7.14)	*N* = 281	8.91(6.00–13.03)	< 0.0001*
Monocytes, % (*N* = 993)	8.10(5.40–10.40)	*N* = 712	8.90(7.00–10.90)	*N* = 281	4.60(2.70–7.50)	< 0.0001*
Neutrophils, % (*N* = 993)	73.60(63.70–83.90)	*N* = 712	69.00(60.30–77.35)	*N* = 281	87.10(79.90–92.20)	< 0.0001*
Eosinophils, % (*N* = 993)	0.30(0.00–1.30)	*N* = 712	0.70(0.00–1.70)	*N* = 281	0.00(0.00–0.10)	< 0.0001*
Immune cell subsets						
Lymphocytes, # (*N* = 993), × 10^9^/L	0.94(0.64–1.34)	*N* = 712	1.09(0.76–1.46)	*N* = 281	0.63(0.44–0.85)	< 0.0001*
CD3^+^CD19^−^ T cells, # (*N* = 207), /μL	782.00(394.00–1060.00)	*N* = 153	905.00(682.00–1173.00)	*N* = 54	276.50(132.75–408.50)	< 0.0001*
CD3^+^CD8^+^ T cells, # (*N* = 207), /μL	248.00(112.50–350.50)	*N* = 153	280.00(206.00–384.00)	*N* = 54	62.00(29.25–127.00)	< 0.0001*
CD3^−^CD19^+^ B cells, # (*N* = 207), /μL	137.00(78.00–205.00)	*N* = 153	156.00(117.00–209.00)	*N* = 54	73.50(40.25–143.00)	< 0.0001*
CD3^−^CD16^+^CD56^+^ NK cells, # (*N* = 207), /μL	160.00(72.50–278.50)	*N* = 153	209.00(128.00–313.00)	*N* = 54	36.50(16.00–74.75)	< 0.0001*
T cells + B cells + NK cells, # (*N* = 207), /μL	1134.00(687.00–1533.00)	*N* = 153	1337.00(995.00–1662.00)	*N* = 54	406.00(269.75–692.50)	< 0.0001*
Inflammatory cytokines and biomarkers						
IL-6 (*N* = 796), pg/mL	14(3.42–49.38)	*N* = 594	6.85(2.82–24.18)	*N* = 202	61.35(29.27–151.45)	< 0.0001*
IL-10 (*N* = 778), pg/mL	5.00(5.00–8.90)	*N* = 579	5.00(5.00–5.85)	*N* = 199	10.30(6.35–18.70)	< 0.0001*
IL-8 (*N* = 779), pg/mL	14.20(8.00–27.95)	*N* = 579	11.60(6.80–20.25)	*N* = 200	28.40(16.35–61.83)	< 0.0001*
TNF-α (*N* = 786), pg/mL	8.60(6.60–11.80)	*N* = 586	8.10(6.10–10.70)	*N* = 200	11.45(8.1–16.50)	< 0.0001*
IL-1β (*N* = 778), pg/mL	6.92(7.44)	*N* = 578	6.49(6.35)	*N* = 200	8.15(9.88)	< 0.0001*
IL-2R (*N* = 774), U/mL	675.50(446.25–1078.00)	*N* = 576	599.50(407.75–873.25)	*N* = 198	1148.00(740.25–1615.00)	< 0.0001*
Ferritin (*N* = 602), μg/L	751.00(421.10–1439.05)	*N* = 420	566.90(351.53–989.13)	*N* = 182	1407.35(832.68–2400.18)	< 0.0001*
hs-CRP (*N* = 980), mg/L	41.05(6.90–98.38)	*N* = 704	20.85(3.58–66.33)	*N* = 276	105.60(59.33–164.43)	< 0.0001*
Procalcitonin (*N* = 890), ng/mL	0.08(0.05–0.21)	*N* = 629	0.06(0.04–0.09)	*N* = 261	0.29(0.12–0.89)	< 0.0001*
Organ damage indexes						
ALT (*N* = 991), U/L	24.00(15.00–39.00)	*N* = 710	23.00(15.00–38.00)	*N* = 281	27.00(18.00–42.00)	0.004*
AST (*N* = 991), U/L	30.00(21.00–44.00)	*N* = 710	26.50(19.00–38.00)	*N* = 281	41.00(29.00–59.00)	< 0.0001*
TBIL (*N* = 993), μmol/L	10.00(7.40–13.90)	*N* = 712	9.40(7.20–12.55)	*N* = 281	12.30(9.00–18.60)	< 0.0001*
ALB/GLO (*N* = 988)	0.99(0.83–1.21)	*N* = 708	1.05(0.89–1.26)	*N* = 280	0.86(0.75–1.00)	< 0.0001*
LDH (*N* = 950), U/L	302.00(225.00–442.75)	*N* = 673	262.00(209.00–328.00)	*N* = 277	504.00(364.00–669.00)	< 0.0001*
ALP (*N* = 952), U/L	68.00(56.00–86.00)	*N* = 671	66.00(55.50–79.00)	*N* = 281	78.00(60.00–107.00)	< 0.0001*
Amylase (*N* = 486), U/L	63.50(43.00–83.00)	*N* = 342	67.00(48.00–83.00)	*N* = 144	52.00(36.00–84.00)	0.004*
eGFR (*N* = 987), mL/(min*1.73 m^2^)	82.60(66.80–92.80)	*N* = 709	85.90(71.60–93.00)	*N* = 278	72.60(47.83–90.98)	< 0.0001*
Creatinine (*N* = 988), μmol/L	78.00(65.00–93.25)	*N* = 712	75.00(64.00–88.00)	*N* = 281	86.00(66.50–114.00)	< 0.0001*
NT-proBNP (*N* = 841), pg/mL	230.00(89.00–744.00)	*N* = 589	152.00(64.00–335.00)	*N* = 252	888.50(362.50–2567.00)	< 0.0001*
CK-MB (*N* = 650), U/L	1.00(0.60–1.90)	*N* = 477	0.80(0.50–1.30)	*N* = 173	2.40(1.20–5.80)	< 0.0001*
hs-cTnI (*N* = 905), pg/mL	7.70(3.60–20.70)	*N* = 644	5.40(2.80–10.93)	*N* = 261	35.30(11.20–194.70)	< 0.0001*
Platelets, # (*N* = 988), × 10^9^/L	206.00(149.00–273.25)	*N* = 712	219.00(167.00–285.00)	*N* = 281	159.00(111.00–223.50)	< 0.0001*
PT (*N* = 980), s	14.10(13.50–15.00)	*N* = 699	13.90(13.30–14.40)	*N* = 281	15.30(14.30–16.90)	< 0.0001*
APTT (*N* = 930), s	39.40(36.10–43.50)	*N* = 680	39.25(36.00–42.83)	*N* = 250	40.25(36.23–46.00)	0.005*
D-Dimer (*N* = 966), μg/mL	1.14(0.51–2.68)	*N* = 691	0.77(0.41–1.64)	*N* = 275	4.10(1.39–15.70)	< 0.0001*
FDP (*N* = 727), g/L	4.50(4.00–14.40)	*N* = 515	4.00(4.00–5.50)	*N* = 212	25.85(7.40–86.50)	< 0.0001*
PTA (*N* = 980), %	87.00(77.75–95.25)	*N* = 699	90.00(84.00–98.00)	*N* = 281	75.00(62.00–86.00)	< 0.0001*
Metabolism indexes						
HbA1c (*N* = 351), %	6.40(6.00–7.30)	*N* = 251	6.30(6.00–7.15)	*N* = 100	6.65(6.10–7.43)	0.022*
K^+^ (*N* = 993), mmol/L	4.23(3.80–4.61)	*N* = 712	4.19(3.78–4.55)	*N* = 281	4.36(3.88–4.86)	0.001*
Ca^2+^ (*N* = 992), mmol/L	2.12(2.04–2.21)	*N* = 712	2.15(2.07–2.23)	*N* = 281	2.06(1.99–2.14)	< 0.0001*
Blood gas characteristics						
PaO_2_ (*N* = 303), mmHg	82.00(68.00–88.00)	*N* = 181	85.70(81.00–92.00)	*N* = 122	68.30(52.30–80.80)	< 0.0001*
SaO_2_ (*N* = 303), %	95.70(88.00–98.50)	*N* = 181	98.10(95.90–99.10)	*N* = 122	85.50(71.00–92.00)	< 0.0001*
PaCO_2_ (*N* = 304), mmHg	37.50(32.28–42.33)	*N* = 182	39.80(34.50–43.35)	*N* = 122	34.60(30.30–37.90)	< 0.0001*
Actual bicarbonate (*N* = 304), mmol/L	23.75(20.50–25.80)	*N* = 182	24.40(22.25–26.15)	*N* = 122	22.30(19.30–24.90)	< 0.0001*
Score prediction	*N* = 303		*N* = 181		*N* = 122	
SOFA score	3.00(2.00–4.00)		2.00(2.00–3.00)		4.00(3.00–6.00)	< 0.0001*
qSOFA score	0.00(0.00–1.00)		0.00(0.00–1.00)		1.00(0.25–1.00)	< 0.0001*
APACHE II score	13.00(9.00–17.00)		10.00(7.00–13.00)		17.00(14.00–20.00)	< 0.0001*
SIRS score	1.00(1.00–2.00)		1.00(0.00–2.00)		2.00(1.00–2.00)	< 0.0001*

*P < 0.05

**Fig. 1:**
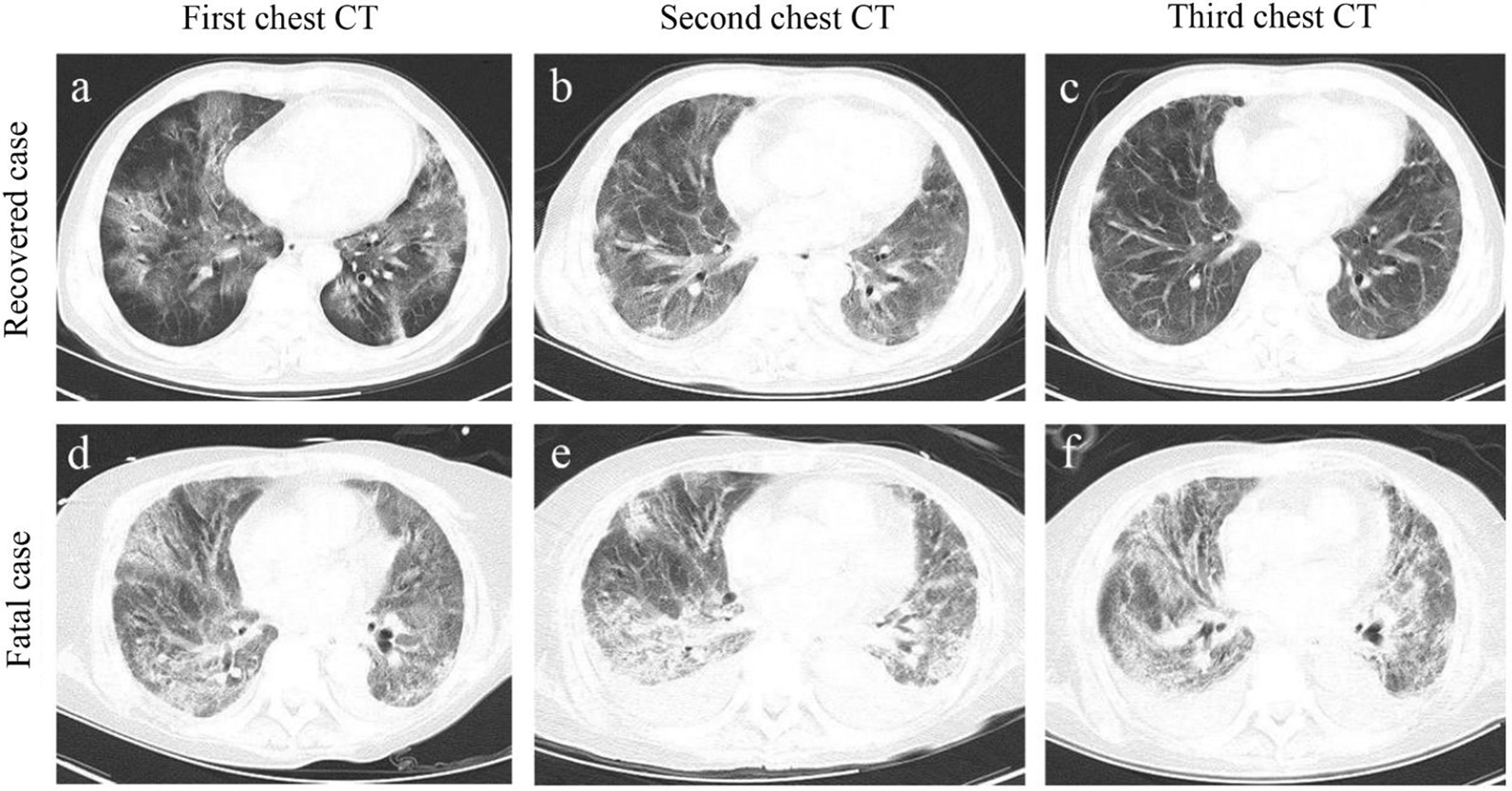
Representative chest computed tomographic images of fatal and recovered patients with COVID-19. a-c. An elderly male recovered case confirmed with COVID-19 at different disease stages. **a** Axial chest CT image obtained at the onset shows diffuse ground-glass opacity (GGO) and fibrous stripes; **b** axial chest CT image obtained at the middle stage shows bilateral, peripheral GGO and fibrous stripes associated with smooth interlobular and intralobular septal thickening; **c** axial chest CT image obtained at discharge stage shows bilateral fibrous stripes and nodules. **d**–**f** An elderly male fatal case confirmed with COVID-19 at different disease stages. **a** Axial chest CT obtained at the onset shows bilateral diffuse GGO associated with round cystic change, local bronchial meteorology and left pleural effusion; **e** axial chest CT image obtained at the middle stage shows the progression of bilateral GGO, bronchial meteorology and pleural effusion; **f** axial chest CT image obtained near stage of death shows the progression of bilateral GGO, round cystic change, bronchial meteorology, and increase of pleural effusion

The abnormalities in chest CT images among these patients were also observed (Table 1 and Fig. 1). The patchy shadows presence was the most typical manifestation of both groups, while pleural thickening (41.38% vs 26.90%; *P* = 0.018) was more frequently observed in non-survivors, compared with survivors. Consistently, blood gas analysis revealed the degree of hypoxia was more evident in non-survivors, which presented lower concentration of PaO_2_, SaO_2_, PaCO_2_, CtCO_2_ and bicarbonate than survivors. These findings suggest more serious impairment of lung function in non-survivors.

Previous studies reported that cytokine storm and lymphopenia were common features in severe COVID-19 patients [2, 15]. We observed that the serum levels of inflammatory cytokines including IL6 (61.35 vs 6.85 pg/mL; *P* < 0.0001), IL10 (10.30 vs 5.00 pg/mL; *P* < 0.0001), IL8 (28.40 vs 11.60 pg/mL; *P* < 0.0001), TNF-α (11.45 vs 8.10 pg/mL; *P* < 0.0001), IL-1β (8.15 vs 6.49 pg/mL; *P* < 0.0001) and IL2R (1148.00 vs 599.50 U/mL; *P* < 0.0001) were significantly elevated in non-survivors compared with survivors. Moreover, the infection-related biomarkers including ferritin, CRP and procalcitonin also exhibited higher levels in non-survivors. Conversely, the baseline counts of lymphocytes (0.63 vs 1.09 × 10^9^/L; *P* < 0.0001), CD3^+^CD19^−^ T cells (276.50 vs 905.00/μL, *P* < 0.0001), CD3^+^CD8^+^ T cells (62.00 vs 280.00/μL; *P* < 0.0001), B cells (73.50 vs 156.00/μL, *P* < 0.0001), NK cells (36.50 vs 209.00/μL, *P* < 0.0001) as well as the total number of T cells, B cells and NK cells (406.00 vs 1337.00/μL; *P* < 0.0001) were drastically decreased in non-survivors, compared with survivors. Collectively, these findings demonstrate that aggravated inflammatory responses and severe lymphopenia might be correlated with the poor clinical outcome of COVID-19 patients.

Multiple-organ damage was more pronounced in non-survivors. We observed higher levels of ALT, TBIL, LDH, homocysteine, NT-proBNP, hs-cTnI, CK-MB and lower level of ALB/GLO in non-survivors. Besides, non-survivors had a falling count of eosinophils while elevated leukocytes and neutrophils, compared with survivors. Of note, in non-survivors, coagulation-related biomarkers of platelets counts were also substantially decreased, followed by the increased D-dimer and prolonged PT and APTT.

Additionally, in term of several scores evaluating disease severity, the fatal cases had higher SOFA (4.00 vs 2.00, *P* < 0.0001), qSOFA (1.00 vs 0.00, *P* < 0.0001), APACHE II (17.00 vs 10.00, *P* < 0.0001) and SIRS scores (2.00 vs 1.00, *P* < 0.0001), compared to recovered patients (Table 1).

### Complications and clinical treatments of non-survivors and survivors with COVID-19

SARS-COV-2 infection can cause both pulmonary and multi-system inflammation, leading critical complications (Table 2). The frequency of acute cardiac injury (79.72% vs 11.80%, *P* < 0.0001), heart failure (71.53% vs 6.32%, *P* < 0.0001), acute kidney injury (48.40% vs 4.35%, *P* < 0.0001), acute liver injury (24.20% vs 2.39%, *P* < 0.0001), acute pancreatic injury (4.27% vs 1.54%, *P* = 0.010) especially acute respiratory distress syndrome (ARDS, 96.80% vs 53.79%, *P* < 0.0001) and disseminated intravascular coagulation (DIC, 19.22% vs 0.56%, *P* < 0.0001) were more higher in non-survivors than survivors. These critical complications could be the main cause of death, and the underlying mechanisms warrant further investigations.

**Table 2:** Complications and clinical treatments of survivors and non-survivors with COVID-19

Indicators	Total	Survivors	Non-survivors	*P* value
	*N* = 993	*N* = 712	*N* = 281	
Complications				
ARDS	655(65.96%)	383(53.79%)	272(96.80%)	< 0.0001*
Acute cardiac injury	308(31.02%)	84(11.80%)	224(79.72%)	< 0.0001*
Heart failure	246(24.77%)	45(6.32%)	201(71.53%)	< 0.0001*
Acute kidney injury	167(16.82%)	31(4.35%)	136(48.40%)	< 0.0001*
Hyperkalaemia	150(15.11%)	38(5.34%)	112(39.86%)	< 0.0001*
Acidosis	105(10.57%)	42(5.90%)	63(22.42%)	< 0.0001*
Acute liver injury	85(8.56%)	17(2.39%)	68(24.20%)	< 0.0001*
Alkalosis	67(6.75%)	44(6.18%)	23(8.19%)	0.256
DIC	58(5.84%)	4(0.56%)	54(19.22%)	< 0.0001*
Acute pancreatic injury	23(2.32%)	11(1.54%)	12(4.27%)	0.010*
Length of hospital stay	18(10–26)	22(15–28)	8(4–14)	< 0.0001*
ICU admission				
ICU	151(15.21%)	4(0.56%)	147(52.69%)	< 0.0001*
Non-ICU	842(84.79%)	708(99.44%)	134(47.31%)	
Treatment^a^				
Antibiotics	894(90.03%)	630(88.48%)	264(93.95%)	0.0094*
Glucocorticoid therapy	754(75.93%)	502(70.51%)	252(89.68%)	< 0.0001*
Antiviral therapy	874(88.02%)	649(91.15%)	225(80.07%)	< 0.0001*
Intravenous immunoglobulin therapy	338(34.04%)	227(31.88%)	101(35.94%)	< 0.0001*
Transfusion	99(9.97%)	21(2.95%)	78(27.76%)	< 0.0001*
Interferon inhalation	353(35.55%)	298(41.85%)	55(19.57%)	< 0.0001*
High flow nasal cannula	241(24.27%)	43(6.04%)	198(70.46%)	< 0.0001*
Mechanical ventilation	365(36.76%)	122(17.13%)	243(86.48%)	< 0.0001*
Non-invasive	311(31.32%)	119(16.71%)	192(68.33%)	< 0.0001*
Invasive	54(5.44%)	3(0.42%)	51(18.15%)	< 0.0001*
Continuous renal replacement therapy	12(12.08%)	2(0.28%)	10(3.56%)	< 0.0001*
Extracorporeal membrane oxygenation	7(0.70%)	1(0.14%)	6(2.14%)	0.0026* ^a^

*COVID-19* Coronavirus disease 2019; *ARDS* Acute respiratory distress syndrome; *DIC* Disseminated intravascular coagulation; *ICU* Intensive care unit

^a^Treatments include antibiotics (cephalosporin, fluoroquinolones, macrolides), antiviral therapy (Lopinavir/ritonavir, ganciclovir, Riboviron, oseltamivir), transfusion (suspended red blood cells, platelets, plasma)

Categorical variables were expressed as number (%). *P* values were calculated by Pearson χ^2^ test, or Fisher's exact test(a). *P < 0.05

Almost all non-survivors received antibiotic treatment (93.95% vs 88.48%, *P* = 0.0094), more than the number of recovered patients (Table 2). Fatal patients received more glucocorticoid therapy, intravenous immunoglobulin therapy or transfusion than recovered cases, since the cytokine storm and DIC were more often observed in non-survivors. Similarly, due to the higher proportions of patients developed acute kidney injury or ARDS in non-survivors, deceased patients received more mechanical ventilation, continuous renal replacement therapy, high flow nasal cannula and extracorporeal membrane oxygenation than recovered patients. These factors might result in more frequent ICU admission (52.69% vs 0.56%, *P* < 0.0001) and shorter time of hospital stay (22[IQR 15–28] vs 8 [IQR 4–14], *P* < 0.0001) in non-survivors than survivors. The clinical interventions were more intensive in non-survivors due to the more severe illness in fatal cases. Of note, recovered patients were undergoing more, antiviral therapy (91.15% vs 80.07%, *P* < 0.0001) as compared to decreased cases.

### Risk factors associated with the death of COVID-19 patients

Furthermore, we performed Cox analysis to identify the potential death risk factors of COVID-19 with adjustment of age, sex, comorbidities. As shown in Table 3 and Additional file 1: Table S2, the higher temperature, faster heart rate and respiratory rate and lower mean arterial pressure were associated with increased risk of death. In terms of laboratory parameters, elevated inflammatory cytokines and infection-related factors, such as TNF-α, IL-6, IL-10, IL-8, IL-1β, IL-2R, ferritin, hs-CRP and procalcitonin were significantly associated with higher death risk of COVID-19. Conversely, the immune cells subsets, such as lymphocytes (HR 0.220, 95% CI 0.161–0.302, *P* < 0.0001), B cells (HR 0.994, 95% CI 0.990–0.998, *P* = 0.002), NK cells (HR 0.985, 95% CI 0.981–0.990, *P* < 0.0001), CD3^+^CD19^−^ T cells (HR 0.996, 95% CI 0.995–0.997, *P* < 0.0001) and CD3^+^CD8^+^ T cells (HR 0.989, 95% CI 0.985–0.992, *P* < 0.0001), were significantly associated with the lower death risk of COVID-19.

**Table 3:** Factors associated with the death of COVID-19 patients

Indicators	Univariable Cox regression		Multivariable Cox regression	
	HR (95% CI)	*P* value	HR (95% CI)	*P* value
Vital signs				
Temperature, ℃	1.340(1.169–1.536)	< 0.0001*	1.332(1.160–1.529)	< 0.0001*
Heart rate, bpm	1.036(1.031–1.042)	< 0.0001*	1.037(1.032–1.043)	< 0.0001*
Respiratory rate, bpm	1.118(1.105–1.130)	< 0.0001*	1.123(1.109–1.136)	< 0.0001*
Mean arterial pressure, mmHg	0.958(0.951–0.966)	< 0.0001*	0.958(0.950–0.966)	< 0.0001*
Laboratory examinations				
Blood routine				
Leukocytes, # (*N* = 993), × 10^9^/L	1.075(1.065–1.085)	< 0.0001*	1.095(1.081–1.109)	< 0.0001*
Monocytes, % (*N* = 993)	0.822(0.793–0.852)	< 0.0001*	0.816(0.787–0.847)	< 0.0001*
Neutrophils, % (*N* = 993)	1.106(1.091–1.120)	< 0.0001*	1.107(1.092–1.121)	< 0.0001*
Eosinophils, % (*N* = 993)	0.298(0.224–0.395)	< 0.0001*	0.301(0.227–0.398)	< 0.0001*
Hemoglobin (*N* = 993), g/L	0.994(0.989–1.000)	0.044*	0.993(0.987–0.998)	< 0.0001*
Immune cells subsets				
Lymphocytes, # (*N* = 993), × 10^9^/L	0.216(0.158–0.297)	< 0.0001*	0.220(0.161–0.302)	< 0.0001*
CD3^+^CD19^−^ T cells, # (*N* = 207), /μL	0.996(0.995–0.997)	< 0.0001*	0.996(0.995–0.997)	< 0.0001*
CD3^+^CD4^+^ T cells, # (*N* = 207), /μL	1.000(0.999–1.001)	0.786	1.000(0.999–1.001)	0.861
CD3^+^CD8^+^ T cells, # (*N* = 207), /μL	0.989(0.986–0.992)	< 0.0001*	0.989(0.985–0.992)	< 0.0001*
CD3^−^CD19^+^ B cells, # (*N* = 206), /μL	0.994(0.990–0.997)	0.001*	0.994(0.990–0.998)	0.002*
CD3^−^CD16^+^CD56^+^ NK cells, # (*N* = 207), /μL	0.986(0.981–0.990)	< 0.0001*	0.985(0.981–0.990)	< 0.0001*
T cells + B cells + NK cells, # (*N* = 207), /μL	0.997(0.997–0.998)	< 0.0001*	0.997(0.996–0.998)	< 0.0001*
Inflammatory cytokines and biomarkers				
IL-6 (*N* = 796), pg/mL	1.001(1.001–1.002)	< 0.0001*	1.001(1.001–1.002)	< 0.0001*
IL-10 (*N* = 778), pg/mL	1.008(1.006–1.010)	< 0.0001*	1.009(1.006–1.012)	< 0.0001*
IL-8 (*N* = 779), pg/mL	1.000(1.000–1.001)	< 0.0001*	1.000(1.000–1.001)	< 0.0001*
TNF-α (*N* = 786), pg/mL	1.055(1.045–1.066)	< 0.0001*	1.059(1.048–1.070)	< 0.0001*
IL-1β (*N* = 778), pg/mL	1.018(1.005–1.031)	0.007*	1.021(1.007–1.036)	0.004*
IL-2R (*N* = 774), U/mL	1.001(1.001–1.001)	< 0.0001*	1.001(1.001–1.001)	< 0.0001*
Ferritin (*N* = 602), μg/L	1.000(1.000–1.000)	< 0.0001*	1.000(1.000–1.000)	< 0.0001*
hs-CRP (*N* = 980), mg/L	1.010(1.008–1.011)	< 0.0001*	1.010(1.008–1.011)	< 0.0001*
Procalcitonin (*N* = 890), ng/mL	1.051(1.036–1.066)	< 0.0001*	1.051(1.036–1.067)	< 0.0001*
Organ damage indexes				
ALT (*N* = 991), U/L	1.001(1.000–1.001)	0.002*	1.001(1.000–1.001)	0.002*
AST (*N* = 991), U/L	1.001(1.000–1.001)	< 0.0001*	1.001(1.000–1.001)	< 0.0001*
TBIL (*N* = 986), μmol/L	1.006(1.004–1.008)	< 0.0001*	1.007(1.005–1.009)	< 0.0001*
ALB (*N* = 988), g/L	0.875(0.854–0.896)	< 0.0001*	0.874(0.853–0.895)	< 0.0001*
LDH (*N* = 950), U/L	1.003(1.002–1.003)	< 0.0001*	1.003(1.002–1.003)	< 0.0001*
ALP (*N* = 952), U/L	1.003(1.002–1.005)	< 0.0001*	1.004(1.002–1.005)	< 0.0001*
Amylase (*N* = 486), U/L	1.002(1.000–1.004)	0.021*	1.002(1.000–1.004)	0.038*
eGFR (*N* = 987), mL/(min*1.73 m^2^)	0.983(0.978–0.988)	< 0.0001*	0.981(0.976–0.986)	< 0.0001*
Creatinine (*N* = 988), μmol/L	1.002(1.002–1.003)	< 0.0001*	1.002(1.002–1.003)	< 0.0001*
NT-proBNP (*N* = 841), pg/mL	1.000(1.000–1.000)	< 0.0001*	1.000(1.000–1.000)	< 0.0001*
CK-MB (*N* = 650), U/L	1.039(1.031–1.047)	< 0.0001*	1.048(1.036–1.060)	< 0.0001*
hs-cTnI (*N* = 905), pg/mL	1.000(1.000–1.000)	< 0.0001*	1.000(1.000–1.000)	< 0.0001*
Platelets, # (*N* = 988), × 10^9^/L	0.994(0.992–0.995)	< 0.0001*	0.994(0.992–0.995)	< 0.0001*
PT (*N* = 980), s	1.046(1.036–1.055)	< 0.0001*	1.045(1.036–1.055)	< 0.0001*
APTT (*N* = 930), s	1.025(1.016–1.034)	< 0.0001*	1.025(1.016–1.034)	< 0.0001*
D-Dimer (*N* = 971), μg/mL	1.129(1.113–1.144)	< 0.0001*	1.131(1.115–1.147)	< 0.0001*
FDP (*N* = 765), g/L	1.018(1.016–1.020)	< 0.0001*	1.018(1.016–1.021)	< 0.0001*
PTA (*N* = 980), %	0.959(0.954–0.964)	< 0.0001*	0.957(0.951–0.962)	< 0.0001*
Metabolism indexes				
K^+^ (*N* = 993), mmol/L	1.538(1.295–1.826)	< 0.0001*	1.540(1.291–1.837)	< 0.0001*
Ca^2+^ (*N* = 992), mmol/L	0.025(0.011–0.058)	< 0.0001*	0.025(0.010–0.058)	< 0.0001*
Blood gas characteristics				
PaO_2_ (*N* = 304), mmHg	0.958(0.949–0.967)	< 0.0001*	0.956(0.947–0.966)	< 0.0001*
SaO_2_ (*N* = 304), %	0.956(0.949–0.963)	< 0.0001*	0.954(0.947–0.962)	< 0.0001*
PaCO_2_ (*N* = 304), mmHg	0.946(0.924–0.970)	< 0.0001*	0.950(0.926–0.974)	< 0.0001*
Actual bicarbonate (*N* = 304), mmol/L	0.922(0.887–0.958)	< 0.0001*	0.930(0.894–0.967)	< 0.0001*
Score prediction				
SOFA score	1.340(1.276–1.408)	< 0.0001*	1.437(1.348–1.532)	< 0.0001*
qSOFA score	3.435(2.577–4.580)	< 0.0001*	3.471(2.600–4.634)	< 0.0001*
APACHE II score	1.172(1.137–1.208)	< 0.0001*	1.195(1.156–1.234)	< 0.0001*
SIRS score	1.854(1.567–2.194)	< 0.0001*	1.924(1.620–2.286)	< 0.0001*
Complications				
ARDS	16.051(8.262–31.186)	< 0.0001*	16.216(8.341–31.527)	< 0.0001*
Acute cardiac injury	11.435(8.543–15.307)	< 0.0001*	13.023(9.678–17.524)	< 0.0001*
Heart failure	10.201(7.860–13.238)	< 0.0001*	10.722(8.227–13.974)	< 0.0001*
Acute kidney injury	5.957(4.707–7.539)	< 0.0001*	6.063(4.759–7.724)	< 0.0001*
Hyperkalaemia	3.999(3.148–5.081)	< 0.0001*	4.041(3.166–5.157)	< 0.0001*
Acidosis	2.658(2.007–3.520)	< 0.0001*	2.621(1.965–3.495)	< 0.0001*
Acute liver injury	4.097(3.114–5.388)	< 0.0001*	4.241(3.212–5.599)	< 0.0001*
DIC	5.682(4.209–7.672)	< 0.0001*	5.819(4.281–7.910)	< 0.0001*

*P < 0.05

Indicators that represented organ damages, such as elevated ALT, AST, LDH, creatinine, amylase, hs-cTnI, NT-proBNP, CK-MB, and decreased ALB/GLO aggrandized the risk for COVID-19 death. Furthermore, coagulation-related biomarkers, including declined platelet counts (HR 0.994, 95% CI 0.992–0.995, *P* < 0.0001), and increased PT, APTT, D-Dimer (HR 1.025–1.359, *P* < 0.0001), might be strong indicators for death risk of COVID-19. Moreover, apart from abnormal biochemical dynamics, metabolism indices and blood gas analysis such as decreased serum level of PaO_2_, SaO_2_, PaCO_2_, and CtCO_2_ could result in the degree of electrolyte disturbance or hypoxia, increasing the risk of death of COVID-19.

We also analyzed the risk of death for patients with complications, patients with ARDS (HR 16.216, 95% CI 8.341–31.527, *P* < 0.0001) had highest risk of death, followed by acute cardiac injury (HR 13.023, 95% CI 9.678–17.524, *P* < 0.0001), heart failure (HR 10.722, 95% CI 8.227–13.974, *P* < 0.0001), acute kidney injury (HR 6.063, 95% CI 4.759–7.724, *P* < 0.0001) and DIC (HR 5.819, 95% CI 4.281–7.910, *P* < 0.0001). Besides, the higher levels of SOFA, qSOFA, APACHE II and SIRS scores were significantly associated with increased death risk (HR 1.195–3.471, *P* < 0.0001). These findings provided evidence supporting that dynamic of inflammatory cytokines, immune cells subsets, blood gas, organ damage biomarkers, and especially complications should be closely monitored, in case of poor outcomes.

### Comprehensive prediction models for death risk of COVID-19 patients

**Fig. 2:**
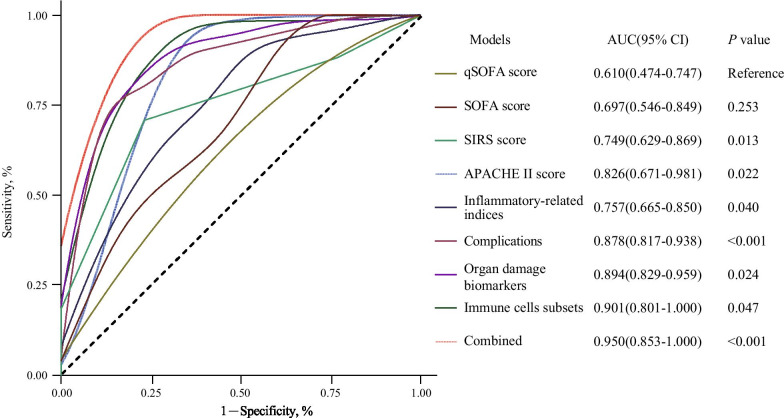
Comprehensive prediction models for death risk of COVID-19 patients. Time-dependent receiver operating characteristic (ROC) curves and area under the curve (AUC) were employed to assess the predictive accuracy of models evaluating the death risk of COVID-19 with SOFA, qSOFA, APACHE II and SIRS scores, inflammatory-related indexes, complications, organ damage indexes, immune cell subsets and combined group integrating abovementioned these factors. The multivariate Cox proportional hazards model analysis were used to establish a risk model. The stepwise regression was used for the selection of the prediction for the model. ROC curves and AUCs (95% CIs) values were generated to assess prognostic accuracy for each model. A two-sided *P* value < 0.05 was considered statistically significant

In light of the SOFA, qSOFA, APACHE II and SIRS scores have been reported as good diagnostic indicators for sepsis, septic shock and multi-organ failure [3, 4, 6–8, 16], we then calculated the prediction accuracy of these four scores in assessing death risk of COVID-19. As presented in Fig. 2, these four scores had prominent prediction capacities evaluating COVID-19 death risk. The AUCs of SOFA, qSOFA, APACHE II and SIRS scores attained 0.697(95% CI 0.546–0.849), 0.610(95% CI 0.474–0.747), 0.826(95% CI 0.671–0.981) and 0.749(95% CI 0.629–0.869). Of note, discrimination of death risk models were better using APACHE II (AUC = 0.826, *P* = 0.022) and SIRS scores (AUC = 0.749, *P* = 0.013) than SOFA or qSOFA scores, which might partly be attributed to aggravated pro-inflammatory responses in non-survivors.

Besides, we also established another four prediction models based on inflammatory-related indices, immune cells subsets, organ damage biomarkers and complications, all of which were significantly associated with the COVID-19 death risk. Among death risk prediction models of each group alone, the predictive accuracy of the immune cells subsets group was the highest (AUC = 0.901, 95% CI 0.801–1.000). Similarly, multiple-organ damage biomarkers (AUC = 0.894, 95% CI 0.829–0.959), inflammatory-related indices (AUC = 0.757, 95% CI 0.665–0.850) and complications (AUC = 0.878, 95% CI 0.817–0.938) had better predictive effects in the discrimination of mortality, outperforming abovementioned SOFA, qSOFA scores (*P* < 0.05) (Fig. 2).

Finally, we integrated four score predictive systems, inflammatory-related indices, immune cells subsets, organ damage biomarkers and complications to construct a combined group. The combined score (AUC = 0.950, 95% CI 0.853–1.000) was significantly higher than that of each risk group alone (Fig. 2), suggesting the combined score system can comprehensively reflect the death risk of COVID-19.

## Discussion

This retrospective large cohort study identified several risk factors and established comprehensive risk models for death in COVID-19 patients. In particular, patients with higher SOFA, qSOFA, APACHE II and SIRS scores, decreased immune cells subsets, elevated inflammatory-related indices, dysregulated multi-organ damage biomarkers and deleterious complications had a higher risk of in-hospital death of COVID-19. Notably, the predictive accuracy of the immune cells subsets group was the highest (AUC = 0.901) among groups alone. Additionally, the predictive accuracy of combining these risk factors (AUC = 0.950) was also significantly higher than that of each risk group alone, outperforming previous risk models.

Our enrolled survivor and non-survivor patients with COVID-19 were statistically matched and excluded the effects of common risk factors, such as age, sex, and underlying comorbidities, on the COVID-19 death, which had been reported to affect COVID-19 mortality [6, 9, 17]. According to the chest CT's of patients, the non-survivors have more deleterious pulmonary invasion. These pulmonary findings provide evidence supporting that the non-survivors are more likely to develop into severe illness. The current study is designed to identify additional potential risk factors and establish a comprehensive evaluation system for the death risk of COVID-19. The SOFA, qSOFA, APACHE II and SIRS scores have been considered as good evaluation predictors for sepsis, septic shock and multi-organ failure [3, 4, 6–8, 16]. Here, we confirmed that those with increased SOFA, qSOFA, APACHE II or SIRS score had a higher death risk of COVID-19. Additionally, the AUC values predicting the death risk of COVID-19 using SOFA, qSOFA, APACHE II or SIRS score attained 0.697, 0.610, 0.826 and 0.749 respectively, consistent with previous reports revealing that these scores are useful predictors of ICU mortality [18–20]. Strikingly, it was reported that SOFA and qSOFA scores had greater capacities predicting death in ICU cohort, compared to APACHE II or SIRS score [20, 21]. However, in the current study, compared to SOFA and qSOFA, APACHE II and SIRS scores are better predictors for COVID-19 death risk and the validation of the scores might been affected by aggravated pro-inflammatory responses in fatal patients.

We found that the serum levels of multiple pro-inflammatory cytokines or biomarkers, including IL-6, IL-10, IL-8, TNF-α, IL-1β, IL-2R, ferritin, hs-CRP and procalcitonin were substantially elevated in non-survivors with COVID-19. Moreover, the increase of leukocytes and neutrophils were also observed in non-survivors. Aggravated pro-inflammatory responses that could lead to increased vascular permeability, extensive pulmonary pathology, ultimately mediating respiratory failure and death[22], which was also found to be a useful predictor evaluating COVID-19 mortality (AUC = 0.757).

Defeating SARS-CoV-2 infection requires well-coordinated innate and adaptive immune responses. Impaired adaptive immune responses may cause uncontrolled inflammatory responses, deleterious tissue damage and even death. Recent evidence has demonstrated that in severe COVID-19 patients, lymphopenia is a common feature, with drastically reduced numbers of CD4^+^ T cells, CD8^+^ T cells, B cells and NK cells [15, 23, 24]. These findings were confirmed in our study, revealing that immune cell subsets, such as CD8^+^ T cells, B cells and NK cells, were drastically reduced in non-survivors, compared to survivors, which is associated with enhanced death risk of COVID-19. Additionally, the AUC value of immune cell subsets that attained 0.901 was highest among risk groups alone. These findings indicated that in death patients with COVID-19, dysregulated immune responses are closely correlated to the poor clinical outcome of COVID-19 and warranted intensively monitor in the clinical treatments.

High levels of pro-inflammatory cytokines and dysregulated immune responses may lead to shock and tissue damage in the heart, liver, kidney and coagulation dysfunction, as well as respiratory failure or multiple organ failure [23]. In the current study, multiple-organ damage biomarkers, such as the substantially elevated concentration of AST, LDH, hs-cTnI, CK-MB, PT-INR, NT-proBNP, PT, APTT, PTA, D-dimer and K^+^, and decreased Ca^2+^, ALB/GLB, eosinophils and platelet counts, were found to be abnormally dysregulated in non-survivors and were significantly associated with increased death risk of COVID-19. Additionally, the patients with lung injury will not only lead to hypoxia, but also inflammatory response, which were more likely to develop complications such as the heart failure, acidosis, acute cardiac, kidney and liver injury, especially ARDS and DIC.. Of note, the COVID-19 patients with complications had a higher risk of developing death, consistent with recent reports which reveal that multiple-organ dysfunction and deleterious complications were more pronounced in severe and fatal COVID-19 cases [5, 9]. Similarly, multiple-organ complications and lung lesions have also been found to be closely associated with poor outcomes in influenza and other respiratory viral infections [25–27]. The organ damage biomarkers (AUC = 0.894) and complications (AUC = 0.878) also presented prominent prediction effects of assessing death risk for COVID-19.

Considering that the indices related to inflammatory responses, adaptive immune responses and multiple organ damage showed great predictive effects of death risk (AUC = 0.950), the allocation of medical resources might be based on these indices. For these areas with more medical resources, inflammatory responses and immune dysfunction control, aggressive supportive medical care or earlier admission to the ICU might be the treatment emphasis for patients with high death risk. To date, no vaccine or specific antiviral treatment for COVID-19 has been widely applied, thus supportive therapy that eases the symptoms and protects against important organ damage may be more beneficial for decreasing the fatality rate of COVID-19.

This study has several limitations. First, given that age, sex, and comorbidities, such as diabetes, hypertension, and coronary heart disease (CHD) have been reported as the universal risk factors of COVID-19 death, these factors were statistically matched between survivors and non-survivors and were not included in this study. Our current study was the largest and the laboratory indices were the most comprehensive in fatal COVID-19 patients worldwide. However, due to the nature of the retrospective study, not all laboratory tests were done in all patients, such as lymphocytes subtypes and arterial blood gas tests. Furthermore, this is a single-center observational study, which needs to be further confirmed in additional validation sets.

## Conclusions

In summary, in this large retrospective cohort study with adequate, standardized and unified data, we identified that higher SOFA, qSOFA, APACHE II and SIRS scores, decreased immune cells subsets, elevated inflammation cytokines, dysregulated multi-organ damage biomarkers and deleterious complications were significantly associated with increased death risk of COVID-19. Finally, we established a combined model, comprehensively integrating immune and inflammatory-related factors and other risk factors, which might be more suitable for death risk evaluation of COVID-19 than previous risk models, such as SOFA and qSOFA scores. Our data may provide critical information for the successful clinical management and health decision-making of COVID-19, especially for severe cases.

## Supplementary Information

**Additional file 1.** Definitions and references of some abbreviations in the article.

## Data Availability

The datasets analysed during the current study are available from the corresponding author on reasonable request.

## Abbreviations

COVID-19: The coronavirus disease 2019
SARS-CoV-2: Severe acute respiratory syndrome coronavirus 2
SOFA: Sequential organ failure assessment
qSOFA: Quick SOFA
APACHE II: Acute physiology, chronic health evaluation II
SIRS: Systemic inflammatory response syndrome
NT-proBNP: N-terminal pro brain natriuretic peptide
CT: Computed tomographic
ARDS: Acute respiratory distress syndrome
DIC: Disseminated intravascular coagulation
IQR: Interquartile range
SD: Standard deviation
HR: Hazard ratios
CI: Confidence intervals
ROC: Receiver operating characteristic
AUC: Areas under the curves
IL6: Interleukin 6
TNF-α: Tumor necrosis factor-α
IL2R: Interleukin 2 receptor
CRP: C-reactive protein
ALT: Glutamic-pyruvic transaminase
AST: Glutamic-oxalacetic transaminease
TBIL: Total bilirubin
LDH: Lactate dehydrogenase
hs-cTnI: High-sensitivity cardiac troponin
CK-MB: Creatine kinase-MB
ALB/GLO: Albumin globulin ratio
PT: Prothrombin time
APTT: Activated partial thromboplastin time
ICU: Intensive care unit
IQR: Interquartile range
CHD: Coronary heart disease
