# Supplementary material for: Clinical characteristics and risk factors of fatal patients with COVID-19: a retrospective cohort study in Wuhan, China
Source: BMC Infect Dis. 2021 Sep 14;21:951. doi: 10.1186/s12879-021-06585-8 (PMC8439538; doi:10.1186/s12879-021-06585-8)
Supplement: Supplementary file 1 — Additional file 1. Definitions and references of some abbreviations in the article. [file 12879_2021_6585_MOESM1_ESM.docx]

**Supplemental Methods**

**Definitions and references of some abbreviations in the article.**

Acute respiratory distress syndrome (ARDS) was defined according to the Berlin definition[1]. Acute kidney injury was identified according to the Kidney Disease: Improving Global Outcomes (KDIGO) clinical practice guidelines[2]. Acute pancreatic injury was defined as any abnormality in amylase[3]. Acute liver damage was diagnosed by progressively elevated levels of alanine transaminase (ALT), aspartate aminotransferase (AST) or bilirubin. If the serum levels of cardiac biomarker high-sensitive cardiac troponin I (hs-cTnI) was above the 99th percentile upper reference limit or new abnormalities were shown in electrocardiography and echocardiography, the acute cardiac injury was defined[4]. Disseminated intravascular coagulation (DIC) was diagnosed by increased levels of platelets, clotting factors, and other blood components[5]. The criteria for discharge were an absence of fever for at least 3 days, substantial improvement in respiratory symptoms and both lungs from chest CT, and two throat-swab samples negative for SARS-CoV-2 RNA obtained at least 24 hours apart [6].

SOFA, qSOFA, APACHE II and SIRS scores were calculated by physiological and laboratory parameters recorded within the first 24 hours after hospital admission. The SOFA score (range, 0 [best] to 24 [worst]) uses 6 criteria: low ventilation (PaO2/FiO2≤400 mm Hg), altered mental status (GCS score≤14), damaged cardiovascular function (mean arterial pressure (MAP)≤70 mm Hg), increased bilirubin (≥20 μmol/L), increased creatinine (≥110 μmol/L), and decreased platelet level (≤150×10^9^/L)[7, 8]. The qSOFA (range, 0 [best] to 3 [worst]) uses 3 indicators: hypotension (systolic blood pressure ≤ 100mm Hg), increased respiratory rate (≥22 breaths/min), and abnormal mental status (GCS score≤14)[9]. The APACHE II score (range, 0 [best] to 71 [worst]) is based on 11 routine physiologic indicators, age (≥45 years) and previous comorbidities[10]. The SIRS score (range, 0 [best] to 4 [worst]) includes abnormal body temperatures (≥38℃ or ≤36℃), increased respiratory rate (≥20/min), increased pulse (≥90/min) and irregular leukocytes count (≥12 × 10^9^/L or ≤4 × 10^9^/L)[11].

**References**

1. Force ADT, Ranieri VM, Rubenfeld GD, Thompson BT, Ferguson ND, Caldwell E, Fan E, Camporota L, Slutsky AS: **Acute respiratory distress syndrome: the Berlin Definition**. *JAMA* 2012, **307**(23):2526-2533.

2. Kellum JA, Lameire N, Group KAGW: **Diagnosis, evaluation, and management of acute kidney injury: a KDIGO summary (Part 1)**. *Crit Care* 2013, **17**(1):204.

3. Lee PJ, Papachristou GI: **New insights into acute pancreatitis**. *Nat Rev Gastroenterol Hepatol* 2019, **16**(8):479-496.

4. Huang C, Wang Y, Li X, Ren L, Zhao J, Hu Y, Zhang L, Fan G, Xu J, Gu X *et al*: **Clinical features of patients infected with 2019 novel coronavirus in Wuhan, China**. *Lancet* 2020, **395**(10223):497-506.

5. Levi M, Scully M: **How I treat disseminated intravascular coagulation**. *Blood* 2018, **131**(8):845-854.

6. Zhou F, Yu T, Du R, Fan G, Liu Y, Liu Z, Xiang J, Wang Y, Song B, Gu X *et al*: **Clinical course and risk factors for mortality of adult inpatients with COVID-19 in Wuhan, China: a retrospective cohort study**. *Lancet* 2020, **395**(10229):1054-1062.

7. Vincent JL, Moreno R, Takala J, Willatts S, De Mendonca A, Bruining H, Reinhart CK, Suter PM, Thijs LG: **The SOFA (Sepsis-related Organ Failure Assessment) score to describe organ dysfunction/failure. On behalf of the Working Group on Sepsis-Related Problems of the European Society of Intensive Care Medicine**. *Intensive Care Med* 1996, **22**(7):707-710.

8. Singer M, Deutschman CS, Seymour CW, Shankar-Hari M, Annane D, Bauer M, Bellomo R, Bernard GR, Chiche JD, Coopersmith CM *et al*: **The Third International Consensus Definitions for Sepsis and Septic Shock (Sepsis-3)**. *JAMA* 2016, **315**(8):801-810.

9. Fernando SM, Tran A, Taljaard M, Cheng W, Rochwerg B, Seely AJE, Perry JJ: **Prognostic Accuracy of the Quick Sequential Organ Failure Assessment for Mortality in Patients With Suspected Infection: A Systematic Review and Meta-analysis**. *Ann Intern Med* 2018, **168**(4):266-275.

10. Knaus WA, Draper EA, Wagner DP, Zimmerman JE: **APACHE II: a severity of disease classification system**. *Crit Care Med* 1985, **13**(10):818-829.

11. **American College of Chest Physicians/Society of Critical Care Medicine Consensus Conference: definitions for sepsis and organ failure and guidelines for the use of innovative therapies in sepsis**. *Crit Care Med* 1992, **20**(6):864-874.

| **Supplemental table 1. Demographic, clinical, radiographic, laboratory findings of survivors and non-survivors with COVID-19** | | | | | | | | |
| --- | --- | --- | --- | --- | --- | --- | --- | --- |
| **Indicators** | **Total** |  | **Survivors** | |  | **Non-survivors** | | P value |
|  | N = 993 |  | N = 712 |  |  | N = 281 |  |  |
| Erythrocytes, # (N = 993), ×10^12/L | 4.13(3.69-4.53) |  | N = 712 | 4.11(3.74-4.50) |  | N = 281 | 4.17(3.60-4.63) | 0.936 |
| Hematocrit, % (N = 993) | 37.00(33.50-40.50) |  | N = 712 | 37.00(33.80-40.20) |  | N = 281 | 36.70(32.70-41.20) | 0.774 |
| Hemoglobin (N = 993), g/L | 128.00(116.00-140.00) |  | N = 712 | 128.00(117.00-139.50) |  | N = 281 | 128.00(112.00-143.00) | 0.707 |
| Basophils, % (N = 993) | 0.20(0.10-0.30) |  | N = 712 | 0.20(0.10-0.40) |  | N = 281 | 0.10(0.00-0.20) | **<0.0001*** |
| Lymphocytes, % (N = 993) | 16.50(9.40-24.40) |  | N = 712 | 20.30(13.60-27.40) |  | N = 281 | 7.10(4.10-12.20) | **<0.0001*** |
| CD3+CD19- T cells, % (N = 207) | 67.76(59.45-75.50) |  | N = 153 | 68.64(61.30-75.61) |  | N = 54 | 63.70(55.08-74.59) | 0.061 |
| CD3+CD4+ T cells, % (N = 207) | 43.06(35.60-48.71) |  | N = 153 | 42.98(37.19-48.23) |  | N = 54 | 45.48(31.35-56.10) | 0.650 |
| CD3+CD4+ T cells, # (N = 207), /μL | 86.98(69.22-354.00) |  | N = 153 | 77.74(68.01-415.00) |  | N = 54 | 170.50(93.25-257.50) | 0.094 |
| CD3+CD8+ T cells, % (N = 207) | 20.25(15.59-26.02) |  | N = 153 | 21.92(17.23-26.65) |  | N = 54 | 14.35(9.94-21.01) | **<0.0001*** |
| CD3-CD19+ B cells, % (N = 211) | 21.60(12.79-28.73) |  | N = 158 | 22.72(13.53-28.47) |  | N = 53 | 20.10(12.31-31.61) | 0.981 |
| CD3-CD16+CD56+ NK cells, % (N = 207) | 14.98(9.20-22.24) |  | N = 153 | 16.13(11.02-23.25) |  | N = 54 | 8.80(5.30-15.88) | **<0.0001*** |
| T cells+ B cells+ NK cells, % (N = 206) | 99.28(98.79-99.57) |  | N = 153 | 99.32(98.88-99.59) |  | N = 53 | 99.08(98.57-99.39) | **0.013*** |
| GGT (N = 989), U/L | 32.00(21.00-55.00) |  | N = 708 | 30.00(20.00-49.00) |  | N = 281 | 39.00(25.00-69.00) | **<0.0001*** |
| DBIL (N = 988), μmol/L | 4.60(3.20-6.43) |  | N = 708 | 4.10(3.10-5.60) |  | N = 280 | 6.15(4.40-9.60) | **<0.0001*** |
| IBIL (N = 985), μmol/L | 5.30(3.90-7.40) |  | N = 707 | 5.10(3.80-7.10) |  | N = 278 | 5.95(4.10-8.40) | **0.001*** |
| ALB (N = 988), g/L | 34.00(30.40-37.40) |  | N = 708 | 35.00(31.70-38.70) |  | N = 280 | 31.10(28.00-34.20) | **<0.0001*** |
| GLO (N = 988), g/L | 33.80(30.18-37.53) |  | N = 708 | 33.00(29.50-36.70) |  | N = 280 | 35.60(31.48-39.05) | **<0.0001*** |
| TP (N = 988), g/L | 67.70(64.10-71.70) |  | N = 708 | 68.10(64.80-71.90) |  | N = 280 | 66.50(61.83-70.83) | **<0.0001*** |
| TBA (N = 569), μmol/L | 3.80(2.10-6.10) |  | N = 392 | 3.80(2.20-5.93) |  | N = 177 | 3.40(1.70-6.50) | 0.249 |
| urea (N = 988), mmol/L | 5.40(4.00-7.60) |  | N = 709 | 4.90(3.80-6.30) |  | N = 279 | 8.30(5.60-12.90) | **<0.0001*** |
| Cholinesterase (N = 512), U/L | 5900.00(4609.75-7272.25) |  | N = 331 | 6519.00(5318.00-7644.00) |  | N = 181 | 4813.00(3868.00-5942.00) | **<0.0001*** |
| α-L-fucosidase (N = 493), U/L | 26.00(20.00-36.00) |  | N = 395 | 24.00(19.00-29.00) |  | N = 98 | 119.00(94.25-161.25) | **<0.0001*** |
| Homocysteine (N = 400), U/L | 1.15(0.99-1.44) |  | N = 252 | 1.10(0.98-1.29) |  | N = 148 | 1.28(1.03-1.92) | **<0.0001*** |
| Uric Acid (N = 988), μmol/L | 263.00(202.00-343.25) |  | N = 709 | 262.00(205.00-336.00) |  | N = 279 | 265.20(194.00-371.50) | 0.607 |
| Myohemoglobin (N = 662), ng/mL | 56.15(35.50-136.30) |  | N = 484 | 45.50(33.00-75.95) |  | N = 178 | 237.20(100.40-1200.00) | **<0.0001*** |
| CK (N = 668), U/L | 75.00(44.75-142.50) |  | N = 476 | 63.00(41.00-110.00) |  | N = 192 | 129.00(69.75-332.75) | **<0.0001*** |
| TT (N = 429), s | 16.90(15.90-18.20) |  | N = 181 | 16.60(15.90-17.50) |  | N = 248 | 17.00(15.80-19.10) | **0.002*** |
| PT-INR (N = 980) | 1.09(1.03-1.17) |  | N = 699 | 1.07(1.01-1.12) |  | N = 281 | 1.20(1.10-1.37) | **<0.0001*** |
| AT: A (N = 726), % | 89.00(80.00-99.00) |  | N = 513 | 91.00(82.00-100.00) |  | N = 213 | 83.00(73.00-94.00) | **<0.0001*** |
| Fibrinogen (N = 928), g/L | 4.86(3.64-6.01) |  | N = 680 | 4.87(3.70-5.95) |  | N = 248 | 4.86(3.22-6.33) | 0.892 |
| Glucose (N = 814), mmol/L | 6.71(5.66-8.96) |  | N = 539 | 6.28(5.40-7.87) |  | N = 275 | 7.84(6.37-11.07) | **<0.0001*** |
| Lactate (N = 117), mmol/L | 2.12(1.50-3.08) |  | N = 40 | 1.55(1.14-2.00) |  | N = 77 | 2.55(1.87-3.37) | **<0.0001*** |
| Na+ (N = 992), mmol/L | 139.20(136.30-141.60) |  | N = 712 | 139.30(136.60-141.40) |  | N = 281 | 138.80(135.40-142.70) | 0.904 |
| Standard bicarbonate (N = 303), mmol/L | 24.10(21.90-25.90) |  | N = 181 | 24.50(23.00-25.90) |  | N = 122 | 23.20(20.80-25.80) | **0.003*** |
| CtCO2 (N = 303), mmol/L | 21.30(18.20-23.20) |  | N = 181 | 21.70(19.83-23.58) |  | N = 122 | 19.50(17.20-22.20) | **<0.0001*** |
| Calcium ion-PH correction (N = 972), mmol/L | 2.34(2.24-2.43) |  | N = 695 | 2.33(2.23-2.43) |  | N = 277 | 2.36(2.27-2.44) | **0.015*** |
| Abbreviation: COVID-19, Coronavirus disease 2019; bpm, Beat per minte; CT, Computerized tomography; CD, Cluster of differentiation; NK cells, Natural killer cells; GGT, Gamma-glutamyl transpeptidase; DBIL, Direct bilirubin; IBIL, indirect bilirubin; ALB, Albumin; GLO, Globulin; TP, Total protein; TBA, Total bile acid; CK, Creatine kinase; TT, Thrombin time; PT-INR, Prothrombin international normalized ratio; AT: A, Antithrombin activity. IQR, Interquartile range. | | | | | | | | |
| Continuous variables were described as median (IQR). P values were calculated by Mann-Whitney U non-parameter test for skewed distributed data. *P < 0.05. | | | | | | | | |

| **Supplemental table 2. Factors associated with the death of COVID-19 patients** | | | | | |
| --- | --- | --- | --- | --- | --- |
| **Indicators** | **Univariable Cox regression** | |  | **Multivariable Cox regression** | |
|  | **HR (95% CI)** | ***P*** value |  | **HR (95% CI)** | ***P*** value |
| Basophils, % (*N* = 993) | 0.047(0.021-0.103) | **<0.0001*** |  | 0.046(0.021-0.101) | **<0.0001*** |
| Lymphocytes, % (*N* = 993) | 0.863(0.846-0.881) | **<0.0001*** |  | 0.862(0.845-0.880) | **<0.0001*** |
| CD3^+^CD8^+^ T cells, % (*N* = 207) | 0.953(0.920-0.986) | **0.006*** |  | 0.956(0.923-0.990) | **0.012*** |
| CD3^-^CD16^+^CD56^+^ NK cells, % (*N* = 207) | 0.941(0.908-0.975) | **0.001*** |  | 0.923(0.891-0.956) | **<0.0001*** |
| DBIL (*N* = 988), μmol/L | 1.009(1.006-1.012) | **<0.0001*** |  | 1.009(1.007-1.012) | **<0.0001*** |
| IBIL (*N* = 985), μmol/L | 1.020(1.013-1.027) | **<0.0001*** |  | 1.021(1.014-1.028) | **<0.0001*** |
| GLO (*N* = 988), g/L | 1.029(1.010-1.048) | **0.003*** |  | 1.026(1.007-1.046) | **0.009*** |
| TP (*N* = 988), g/L | 0.946(0.928-0.964) | **<0.0001*** |  | 0.945(0.927-0.964) | **<0.0001*** |
| ALB/GLO (*N* = 988) | 0.120(0.070-0.205) | **<0.0001*** |  | 0.119(0.069-0.206) | **<0.0001*** |
| urea (*N* = 988), mmol/L | 1.097(1.085-1.109) | **<0.0001*** |  | 1.097(1.085-1.110) | **<0.0001*** |
| α-L-fucosidase (*N* = 493), U/L | 1.015(1.013-1.017) | **<0.0001*** |  | 1.016(1.014-1.018) | **<0.0001*** |
| Uric Acid (*N* = 988), μmol/L | 1.002(1.001-1.003) | **0.001*** |  | 1.002(1.001-1.002) | **0.001*** |
| Myohemoglobin (*N* = 662), ng/mL | 1.002(1.002-1.002) | **<0.0001*** |  | 1.002(1.002-1.003) | **<0.0001*** |
| CK (*N* = 668), U/L | 1.001(1.001-1.001) | **<0.0001*** |  | 1.001(1.001-1.001) | **<0.0001*** |
| TT (*N* = 429), s | 1.012(1.002-1.022) | **0.021*** |  | 1.011(1.001-1.022) | **0.027*** |
| PT-INR (*N* = 980) | 1.362(1.267-1.465) | **<0.0001*** |  | 1.359(1.262-1.462) | **<0.0001*** |
| AT: A (*N* = 726), % | 0.964(0.955-0.973) | **<0.0001*** |  | 0.963(0.954-0.972) | **<0.0001*** |
| Glucose (*N* = 814), mmol/L | 1.086(1.064-1.108) | **<0.0001*** |  | 1.100(1.079-1.122) | **<0.0001*** |
| Lactate (*N* = 117), mmol/L | 1.131(1.047-1.222) | **0.002*** |  | 1.147(1.022-1.286) | **0.019*** |
| Na^+^ (*N* = 992), mmol/L | 1.036(1.012-1.062) | **0.003*** |  | 1.040(1.015-1.065) | **0.001*** |
| Standard bicarbonate (*N* = 304), mmol/L | 0.914(0.872-0.958) | **0.0002*** |  | 0.919(0.875-0.965) | **0.001*** |
| CtCO_2_ (*N* = 303), mmol/L | 0.912(0.875-0.951) | **<0.0001*** |  | 0.923(0.884-0.963) | **<0.0001*** |
| Calcium ion-PH correction (*N* = 303), mmol/L | 2.540(1.090-5.921) | **0.031*** |  | 2.654(1.128-6.244) | **0.025*** |
| Acute pancreatic injury | 1.767(0.991-3.151) | 0.054 |  | 1.803(1.008-3.225) | **0.047*** |
| Abbreviation: COVID-19, Coronavirus disease 2019; HR, Hazard ratio; CI, Confidential interval; CD, Cluster of differentiation; NK cells, Natural killer cells; DBIL, Direct bilirubin; IBIL, indirect bilirubin; ALB, Albumin; GLO, Globulin; TP, Total protein; TBA, Total bile acid; CK, Creatine kinase; TT, Thrombin time; PT-INR, Prothrombin international normalized ratio; AT: A, Antithrombin activity. | | | | | |
| HRs and 95% CIs were calculated by univariable and multivariable Cox regression models. *P < 0.05. | | | | | |
